# Hospital and Institutional News

**Published:** 1915-12-18

**Authors:** 


### HOSPITAL AND INSTITUTIONAL NEWS.

### A NEW YORKSHIRE INFIRMARY.

A certain interest attaches to all new institutions for other than war purposes which succeed in becoming opened during the progress of the war. Thus the Brigg Board of Guardians are to be congratulated on the latest addition to Yorkshire institutions which is formed by the opening of their new workhouse infirmary. The old institution has done duty for thirty-four years under the medical officer, Dr. Goodall, who knows only too well the difficulties of inadequate accommodation, and now believes, with the Rev. G. Godfrey, chairman of the Board, that the needs of the sick poor will be satisfied for a considerable time to come in the new building. The architect is Mr. Butterick.

### AN INSTITUTIONAL WILL.

Deeside charities have received some very handsome bequests by the will of Lord Armitstead, particulars of which have just been announced. Thus the Dundee Royal Infirmary gets £10,000; University College, Dundee, £5,000; Dundee Orphan Institution, Sidlaw Sanatorium, Dundee Convalescent Home, *Mars* Training Ship, and Dundee Institution for the Education of the Deaf and Dumb, £1,000 apiece; Dundee Charity Organisation Society, Aged Christian Friends Society of Scotland (Dundee Auxiliary), and Dundee Sick Poor Nursing Society, £500 each. In addition to these munificent legacies, the residue of the estate will ultimately revert to Dundee institutions and charities, which will then benefit to the extent of between £40,000 and £50,000. The strong local patriotism displayed by the testator is a feature which has been noted before in generous Scottish benefactors to voluntary charities.

### MEDICAL INSPECTION IN WAR-TIME.

At a meeting of the Wilts General Education Committee a report was received from the Children's Care Committee on a new, modified scheme of medical inspection to be adopted during the war. Owing to the absence on military service of various medical men, for whom no suitable substitutes could be found, the care committee proposed an emergency plan, the main line of which was the discontinuation of formal inspection and of the collection of statistics, and the development of ameliorative work. This means that new scope will be given to the school nurse; that dental work will continue; that the services of oculists be secured at fees appointed by the committee, while the selection of cases will rest with the school medical officer, and be based on reports from the care committee and nurses. Medical inspection will be arranged when sufficient work accumulates in any district. By these and similar means it is hoped to reduce the cost of the work by one half—from £1,400 to £700.

### TRENCH FOOT AGAIN.

The severe weather in November was accompanied by an outbreak of "trench foot" on a scale which has caused some disappointment to those who confidently expected that the preventive measures adopted would result in the disappearance of this disabling condition. According to an answer in the House of Commons, 770 cases were reported in the week ending November 27, notwithstanding the provision of rubber thigh boots for the men in the trenches. Officially, the large number of cases is ascribed to neglect by the men of the precautions inculcated; and anyone who knows the little ways of Thomas Atkins can well believe that this has been a contributory factor. At the same time, it is too much to hope that any preventive measures will entirely abolish "trench foot"; but they should at least ensure both a diminution of the number of cases and early attention to those that do occur, with correspondingly better results from treatment.

### WOMEN MOTOR AMBULANCE DRIVERS.

The Metropolitan Asylums Board is about to experiment with women drivers for some of their motor ambulances, owing to the shortage of efficient male drivers, a large number of whom have joined the Mechanical Transport branch of the Army Service Corps. Notwithstanding all the efforts of the committee which controls this ambulance service, including the teaching of twenty-three men taken from other departments on condition that they remain at least two years in their new jobs, the service is twenty-one short of the nominal number of drivers; and many of those still driving are attested men who are liable to be called up at any moment. It has, therefore, been decided to staff the North-Western station entirely with women drivers; the men now employed there will be transferred to other stations where there are vacancies. The Women's Reserve Ambulance Corps are co-operating in the provision of skilled women drivers; and there will probably be no great difficulty in obtaining the numbers required. The women drivers will be employed upon the same terms and conditions as men.

### IMPORTANT MASSAGE DEPARTMENTS.

We welcome the following interesting letter from Mr. G. Q. Roberts, Secretary of St. Thomas's Hospital:—"In The Hospital last week, on page 221, under the heading of 'Massage at Manchester,' you produce what is, I think, a mistake. The Manchester paper to which my attention was drawn had your paragraph in, beginning 'The massage department in connection with St. Bartholomew's Hospital, etc.' Is there such a large department at 'Bart.'s'? If so, it is quite right that they should have every credit for it. It is rather strange that the week before this article appeared in the Manchester paper the lady in charge of our massage department at St. Thomas's Hospital was invited to go to Manchester to explain to those, many of whom will work in this new hospital, what can be done with massage as exemplified in the work which has been done at this hospital. So successful was the lecture that so much pressure was brought to bear on her that she was obliged to go across to

Dublin to give a somewhat similar lecture, which again met with great success." In congratulating St. Thomas's Hospital, we may state, in answer to Mr. Roberts' question, that there is a fully equipped massage department at St. Bartholomew's Hospital in association with the physical exercises department, which together constitute a most useful branch of the work done at that important institution.

### THE "LADY SOLDIER" ON THE ENGLISH GENIUS.

Dr. Louisa Garrett Anderson, in speaking at the annual meeting of the Saxmundham War Hospital Supplies Depôt, said that, having gone to France as a member of the Women's Hospital Corps, she had acquired some expert knowledge of the requirements of the wounded. She had been, in fact, what the poor children in the neighbourhood of her hospital called "a lady soldier." Her experience had led her to believe that we, as a nation, had a genius which appeared in voluntary organisations rather than in the official ones. What was wanted of hospital supply depôts, such as that at Saxmundham, was to get straight to the medical officers struggling with serious cases, who often had very little in the way of surgical materials, and to send their goods to hospitals in Serbia or France, and not to those in England. It is very instructive, and also encouraging, for the workers in these supply depôts to hear at first hand from such observers as Dr. Garrett Anderson how best to utilise their goods, and of what value they are to the wounded. The English genius for voluntary organisation has registered in these depôts another success.

### PERMANENT BUILDINGS AT £29 PER BED.

In our issue of December 4 appeared a brief account of the four new pavilions which have been added to the Sunderland Royal Infirmary from Dr. William Robinson's designs. On the basis that each pavilion (which accommodates six patients) costs £600, the cost appeared to work out at £100 per bed. From information now at our disposal it will be seen that £600 is the total cost of the four pavilions, by which the cost per bed worked out at £25 per bed. As a fact, however, the cost of electric light, hot and cold water, curtains and linoleum raised the total to £702, or £29 5s. per bed. As even the latter figure seems remarkably low, it is interesting to examine how it is arrived at. So we append the details of the expenditure. The cost is given as follows: For buildings, £400; twenty-four bedsteads, £60; bedding, dressing-gowns, etc., £116; linoleum, £21; twenty-four cupboards, £22; lavatory basins and hot and cold water supply, £20; lockers and chart-holders, £20; chairs, £3; blinds, £6; screens, £8; hardware (twenty-four copper foot-warmers and two dinner carriers), £11; electric lighting, £20. Total cost for twenty-four beds, £702; cost per bed, £29 5s. It only remains to add that the new pavilions are intended to be permanent structures.

### INFANT CARE AT LEEDS.

Leeds is fortunate in the possession of a Babies' Welcome Association, consisting of a centre and seven branches, for the development of infant care in that city. We have no doubt in our own minds that this Association does much good work, and that it is a blessing to hundreds of infants in Leeds whose lot in life is greatly improved in consequence. But that one of its various energies of which we are immediately cognisant is somewhat misdirected. It consists of a huge chart designed to show graphically the weekly increase in weight of 2,345 infants between birth and a year old. The labour involved in the production of this chart we can well believe to have been colossal; and the expense has been so great that the price is fixed at five shillings per copy. Where the idea seems to us faulty is that the chart conveys nothing useful to any human being, and is simply a waste of much time and effort. The 33,048 weighings which are graphically represented by this chart could, we think, have been made to yield their lessons and their information (though we doubt if either would be novel) by some much simpler and cheaper sort of chart. As it is, one can only deplore that the Leeds Association has not diverted the labour of some of its members into more useful channels.

### THE SCRUPLES OF THE LOCAL GOVERNMENT BOARD.

It would appear as if the Camberwell Board of Guardians were to be faced with what might be termed "a hardy annual," in order that they may adequately remunerate the services which Dr. W. J. C. Keats, their medical superintendent, renders to the borough. Their sole desire was to increase his salary by £50 a year, but, as the Local Government Board declined to sanction this increase, they were obliged to grant Dr. Keats an extra £75 a year in respect of his work in connection with the Old Age Pensions and National Insurance Acts. In granting this extra sum the Board feel they are repaying him some part of the extra salary they have been debarred from granting him for some years. The unfortunate part of the whole matter is that these annual grants lead to continual bickerings, whereas if the additional salary had been granted the matter would have been done with. No member of the Board begrudges Dr. Keats the remuneration, but with the more conscientious members there is a feeling that this is "a back-door" method of getting over the scruples of the Local Government Board. Why, therefore, should the Guardians not make another attempt to induce the Local Government Board to sanction an increase of salary?

### UNFIT RECRUITS.

Even before the final spurt of Lord Derby's recruiting scheme, which must have entailed enormous work on medical inspectors of recruits, there were grumbles to be heard as to the relaxation of the medical standard, officially encouraged and even enjoined. We fear that if this has actually been the case, the recruiting scheme will have enlisted a large number of crocks and chronics whose presence will do the Army nothing but harm. Not long ago a story appeared in the papers to the effect that a medical officer in charge of troops was confronted with a case of valvular disease in a not long enlisted recruit. He began a tirade about the wickedness of passing such a candidate, only to learn that it was he himself who had done it. Another letter which has appeared (in the *Sunday Times*) roundly asserts that glaringly unfit recruits are being accepted, and suggests that doctors who pass them should be heavily fined. We doubt if the proposal is practicable, though we are quite in sympathy with it. On the whole, however, it is satisfactory to note that Lord Derby's recruits are described as being fine men physically: it is doubtless only a small minority that should have been rejected and were not. Still, that small percentage is a serious blot on the reputation of the medical inspectors of recruits, or on those who have forced their hands.

### A NEW WELSH WAR HOSPITAL.

The wish of the original promoters of the Welsh War Hospital, which has been established successfully at Netley, to send a Welsh Hospital to the armies overseas is to be granted. The Army medical authorities now intend to send a Welsh Hospital to the armies in the field, but the new institution, unlike that constructed and maintained at Netley by the generosity of Welshmen, will be established by the War Office. Wales, nevertheless, is to give its name to the new institution, and the people of Wales are to be asked to continue the maintenance of the Netley institution. Dr. Sheen, the administrative chief at Netley, and his principal assistants are expected to form the nucleus of the new hospital's staff, which, presumably, will also be largely drawn from Welshmen. Just as the Welsh Hospital at Netley has the proud claim of being the first voluntary hospital to be presented to the War Office since the war began, so the new institution will hold a special place amongst the oversea institutions.

### THE NOTIFICATION OF MEASLES.

At last the constantly reiterated demands of public health authorities have moved the Local Government Board to stir; and measles (with German measles) is now a notifiable disease throughout England and Wales. Local authorities have adopted a similar measure for their own districts from time to time, but now complete uniformity will be obtained by the Board's Order, and not before it was high time. The Order also enables local authorities to provide medical, including nursing, assistance for those suffering from these diseases. In issuing the Order the Board discuss the disease, its mortality and consequences, and append to the memorandum a leaflet of advice for distribution to parents, emphasising the importance of the disease. Stress is laid upon the saving of child life now sacrificed to this disease; twelve thousand deaths result from it every year. And it is impressed upon local authorities that there is need for taking all practicable steps to safeguard the health of young children.

### THE COST OF BUTTER AND MILK.

There is no doubt that most of our hospitals have now realised the need for very strict economy. At one large hospital it is within our knowledge that recently margarine has been substituted for butter in the daily diets for all who live on the premises; officers and staff as well as patients. The cost of the margarine is 9d. per lb., whereas the butter formerly used cost 1s. 6d. per lb. (contract price). The saving per week under this one heading is over £7. The strange part of the whole thing is that not more than half-a-dozen individuals among the 350 persons who consume the margarine daily are aware that any change has been made. It seems, too, that there is again to be a very decided and alarming advance in the price of milk very soon. One large hospital in the London suburbs makes a yearly contract for milk, and the price now being paid is 11d. per imperial gallon. The present contract expires at the end of this month (December 31.) Some inquiries have been made with reference to the probable quotations for the next contract, and it would appear that 1s. 6d. or even 1s. 8d. per imperial gallon is the figure likely to be quoted.

### THE BILLETING OF TROOPS IN RURAL AREA

Co-operation between the military authorities and local medical officers of health in regard to the billeting of troops was urged on both sides with apparent unanimity at the beginning of the war. In actual practice, however, consultations between the local health officials and Army officers on the subject of the suitability or otherwise of proposed billets were not nearly so frequent as they should have been. In many instances no communications were ever sent by the military to the M.O.H., and sometimes advice tendered was disregarded. The county medical officer for the Soke of Peterborough, in his latest report, refers to instances where soldiers were billeted in houses containing consumptive patients, and even in the tuberculosis dispensary itself! In a small village four men were billeted in a garret which had no window opening to the external air, and cases of overcrowding were not uncommon. Fortunately, a proposal to billet troops in a village where the sanitary conditions are deplorable was nipped in the bud after representations by the local M.O.H. But it is not encouraging to keen medical officers to find themselves ignored by the military authorities in regard to matters on which expert advice is highly desirable.

### MUNICIPAL GRANTS TO VOLUNTARY HOSPITALS.

In certain important centres, such as Liverpool, it has been the custom for local authorities to provide a grant to one or more of the voluntary hospitals. Of late years, of course, the work demanded of the hospitals, such as that relating to the treatment of school children, has made the moral claim of the hospitals to generous treatment stronger than ever. One of the ways in which municipal authorities may be most usefully influenced, and brought to appreciate the public value of hospital work, is through the medium of an active health committee. Thus we find that the Birmingham Maternity Hospital, an institution with twenty-five beds, has approached the Health Committee with the object of persuading the Council to make a grant towards the hospital's expenditure on maternity and infant welfare. The Health Committee is presumably aware not only of the importance of such work, but also of its quality in the present case, and has recommended the City Council to make an annual contribution of £1,500 to the hospital. The recommendation that such a grant should be made is accompanied by the suggested promise that twenty beds should be set aside for difficult cases. That, at least, is the main condition, and while it is proposed that the grant should be made for seven years, the Local Government Board would, it is understood, provide half the money.

### THE LORD MAYOR OF LEEDS AND ITS HOSPITALS.

The institutional life of Leeds is gaining, as it was bound to do, from the fact that Mr. Charles Lupton, treasurer of the General Infirmary, is Lord Mayor of the city. We have made mention of his work for the soldiers, his share in securing the new war hospital provided for them; but the normal institutions of the city are also receiving the benefit of his driving force and practical knowledge. In opening the "Christmas Market" arranged by the Invalid Children's Aid Society, Mr. Lupton pointed out the number of tuberculosis children who were always coming to the infirmary. He added that it was impossible for the hospital to keep them as long as was necessary, and commended the work of the Invalid Children's Aid Society on the ground that it did what it could to supplement the insufficiently prolonged treatment which was all that the hospitals were able to supply. Sir Berkeley Moynihan, who presided over the gathering, pointed out that tuberculosis sprang from over-crowding and its attendant evils, and that after the war the campaign against consumption would have to be renewed with vigour. Sir Berkeley referred to Mr. Lupton's brilliant record of long service and administrative ability.

### GLASGOW CORPORATION'S STRANGE PROPOSAL.

It is hardly surprising that the proposal of the Glasgow Corporation to reduce by one-half its contributions to local hospitals and charities has produced amazement among the institutions. In fact the Corporation has received a deputation from the three large infirmaries on the subject. The chairman of the Royal Infirmary, Mr. James Macfarlane, pointed out the increased needs of the hospitals, their increased work and additional expenses at the present time. He then reminded the Corporation that, before the Corporation's grants were given to the infirmaries, they had received their water supply free; when the charge for water was imposed grants were made from the Common Good, though the grants were insufficient to meet the water charges. He hoped the Corporation would set an example to the citizens by increasing rather than diminishing the present grant. The deputation was thanked for attending, and meanwhile the matter rests. It is hardly conceivable, however, that the Glasgow Corporation, if in search of new economies, could take the fatal step of withdrawing support from the voluntary hospitals, which do such an immense work in checking disease and maintaining health and medical education. To cripple existing hospitals and to start municipal institutions, the logical alternative, would prove about the most expensive change that they could make in Glasgow, to say nothing of its evil effects for the citizens.

### THE NEW SUPPLY DEPOT AT BRADFORD.

With the opening of the new War Hospital Supply Depôt, Bradford gains an institution which is a remarkable example of the value of co-operation in securing large and practical results. Affiliated with the Central Depôt Surgical Branch of the Queen Mary's Needlework Guild, and modelled on the plan of the St. Marylebone War Hospital Central Supply Depôt, its object is to supply the requirements of the Bradford War Hospital at Horton Green. The new depôt has started off with a register of nearly 150 women workers and with a staff nine of whom have received training at the central institution in London. Not only does each worker guarantee certain hours per week, but a subscription of 1s. a month is given by each worker towards the running expenses. That these are likely to be low, thanks to the co-operation of many agencies, may be judged from the following facts. The premises have been lent by the Corporation Street Improvement Committee; the electric-lighting plant has been installed by one firm, the heating apparatus for the passages by another; cupboards and chests have been lent by the Belgian Institute, and so on. Work proceeds from 10 a.m. to 1, and from 2.30 to 6.30. The workers are supplied with tea at 3d. a head. The hon. organising secretary is Miss Laura Pesel. The premises are in Leeds Road. By co-ordinating hours of work, it is hoped to have 100 workers employed throughout the day.

### THE CORONER AND ECONOMY.

At a recent inquest the Coroner for the City and Southwark, one of the most active and many-sided of those who hold this office in London, commented on the fatuousness of holding an inquest without ordering a *post-mortem* examination also. It seems that local authorities have proposed as a war economy that these examinations should be dispensed with whenever possible. We agree with this view, for peace as well as for war time; but the difficulty lies in deciding when an autopsy is necessary and when it is not. Dr. Waldo orders one in 99 per cent. of his inquests, and is convinced that the latter would be farces if the examination were omitted. No doubt he is right in his contention that in the majority of cases autopsy is really indispensable, though we should not have thought the percentage quite as high as he puts it. We know of a case in which a former London Coroner ordered a *post-mortem* on a man whose head had been crushed to pulp by a brewer's dray; and there must be a certain number of such cases where the cause of death is obvious. Incidentally Dr. Waldo is in favour of a small economy by reducing the juryman's fee of two shillings to its ancient level of fourpence; we confess we cannot follow him thus far, though we sympathise with every legitimate economy.

### AN ANTIPODEAN INNOVATION.

A bold step forward in social legislation has just been taken in New Zealand, the practical working of which will be watched with great interest by reformers and hygienists all over the civilised world. An Act has been passed, known as the "Prisoners' Detention Act," dealing with all inmates of prisons who are certified to be suffering from venereal disease. The Governor has power to declare any hospital or part of a hospital, or any part of a prison or gaol, to be a "prison hospital," and to detain there on a Magistrate's order any infected prisoner. There is a clause empowering the authorities, if necessary, to detain such a prisoner for a longer period than the sentence imposed for his offence; that is, until a cure of his disease has been effected. The procedure is to be that any prisoner found to have venereal disease will be brought before a Magistrate in Chambers, with a right of appeal to a Judge of the Supreme Court in Chambers. This avoids publicity, which it is no part of the object of the law to inflict. The object aimed at is the stamping out of venereal disease; and although this legislation touches the fringe only of the evil, still it is a step in the direction of controlling these preventible scourges of our peoples. It is noteworthy that in ultra-democratic New Zealand a law has been passed which would certainly arouse the fiercest opposition in this country among those to whom the "liberty of the subject" is of more importance than the well-being of the community.

### THE BILLETING SYSTEM AT BOURNEMOUTH.

The presidential address lately given by Sir James Crichton Browne at the Conference of the Sanitary Inspectors' Association, which was held at Bournemouth, was of interest for the account of the billeting system that it contained. It seems that the scheme adopted reposes upon the following main lines. A list of unsuitable houses in the different districts has been sent from time to time to the billeting officer, and thus a check has been provided and a standard maintained. It was also found in practice desirable to secure that in houses partly occupied by soldiers, the men should occupy the ground floor and the civilians the rooms overhead. In cases where houses were entirely occupied by troops periodical inspections were made. When an infectious civilian case was removed the military sanitary authorities were notified, and the men were confined to their billets until the military medical officer had paid a visit to them, and the necessary isolation of contacts had been supervised. Moreover, four disinfection stations were established, the food supplies were regularly inspected, refuse collected daily, and additional sanitary officers were installed in the centres in which large numbers of men had been billeted. In other words, the sanitary organisation of Bournemouth rose to the occasion so well that the experiment of billeting troops in the town, where much heart-searching and fears of results unfavourable to its health record were entertained, is to be repeated. As the prosperity of Bournemouth mainly depends upon its reputation for salubrity, its citizens may be forgiven for their anxiety lest the billeting should be followed by an epidemic. Sanitation here, as among the troops in France, has achieved great things.

### THE WELSH COMMISSIONERS AND A PANEL DOCTOR.

The Glamorganshire Insurance Committee have registered a protest against the Welsh Insurance Committee for overriding the finding of the Medical Service Sub-Committee in respect of a complaint made against Dr. Rice Rees, of Ferndale, concerning the issue by him of a certificate relating to a patient whom he had omitted to examine on the occasion when the certificate was signed. It appears that the Commissioners instituted police-court proceedings against a patient for obtaining medical benefit while remaining at work, and as a result they directed that an investigation should be made by the Insurance Committee. The practitioner, when called before the Medical Service Sub-Committee, admitted that on the day when he granted the final certificate he handed it to the patient's wife, without examining the man himself. The sub-committee found that he had done wrong by so acting, apparently at the instance of the patient's wife, and that in future he must confine his certificates to facts of which he was cognisant. The Insurance Committee approved this finding, but the Insurance Commissioners have written, emphasising the serious breach of the regulations involved by the practitioner's action, and stating their decision to short-issue the Insurance Committee to the extent of £20 during the current medical year. The Commissioners asked the committee to take this short-issue into consideration when making any further payments to Dr. Rees in respect of his services to insured persons. The committee were so indignant at the receipt of this letter that the chairman and other members declared they would immediately resign. A vigorous protest to the Commissioners was then agreed on.

### A DOCTOR SUES FOR HIS SALARY AS HOUSE SURGEON.

Cases of medical men suing institutional authorities for salaries are sufficiently rare to make the following Irish case worth quoting. Dr. Nathaniel Mayne has applied in the King's Bench Division, Dublin, for a writ of *mandamus* directing the members of the Joint Committee of the Longford Infirmary to sign a certificate for the payment to him of the salary due to him in his capacity of surgeon to the county infirmary. In 1877 Dr. Mayne was elected surgeon at a salary of £94, with a free house and free fire and light, which last emoluments he subsequently commuted for £30 a year. Up to June 30 last, his counsel explained, the doctor was paid quarterly by the County Council on the production of a certificate signed by five of the infirmary governors. In May last he obtained the certificate signed by five members, one of whom signed outside and not at the meeting. Since then, it was stated, he had not received his salary. He sued the County Council, and the County Court judge gave him a decree for the amount claimed, but this decree was reversed on appeal, partly on the ground that the five members did not sign at the meeting, and that the certificate was not in accordance with Section 36 of the Grand Jury Act of 1836. Application was made for the certificate later, but there had not been a quorum at the meetings summoned, and the certificate had not been signed. The Court adjourned the application, directing that an affidavit, specifying the names of the members of the Joint Committee to whom the *mandamus* should be addressed, should be filed.

### A SUGGESTED AMALGAMATION SCHEME AT PLYMOUTH.

A pessimistic atmosphere prevailed at the recent annual meeting of the Royal Albert Hospital, Devonport, when the disclosure of a serious financial position led to much talk of municipalisation, or closing the institution within twelve months. It is difficult to listen to such talk without impatience, when the very report which gave rise to it contained figures showing that the annual grant to the hospital from the Sunday Fund had increased, notwithstanding the appeals to which the war has given rise. That is enough to show that what is needed is a little more energy, and we therefore welcome the speech made by Mr. K. Eyton Peck, who said that he thought a very false impression might be created by the annual meeting in the town. If they had to close down, Mr. Peck proceeded, the life of another institution, whose financial difficulties were quite as great, would not be long. Large sums had been raised in Plymouth by "proper appeals in the right quarters," and he thought an effort to increase their income should be made. That strikes the right note and shows an appreciation of the true position, which has gone from bad to worse simply by want of driving force and initiative. There is, however, the suggestion of an alternative proposal, endorsed by the Mayor, Mr. T. Baker, for the amalgamation of the Royal Albert Hospital with another institution in the town. From what Mr. Peck said about the financial position of "another institution," we fear that the amalgamation scheme cannot be regarded as likely to solve the lack of income of which the Plymouth hospitals now complain. What is wanted, in fact, is no elaborate contrivance for reducing necessary expenditure, but sufficient energy to secure needed funds. The income of a voluntary hospital is in direct proportion to the amount of intelligence and trouble devoted to maintaining public interest. That a fine field awaits such an attempt is evident from the sums raised for Red Cross purposes in the town.

### LEEDS' LATEST HOSPITAL SCHEME.

With so competent a hospital authority as Mr. Charles Lupton occupying the post of Lord Mayor of Leeds, it was inevitable that the city's arrangements for dealing with the wounded would be better co-ordinated, and on more ambitious lines. An important step has been taken by the decision to extend the 2nd Northern General Hospital at Beckett's Park. A few weeks ago Colonel Littlewood, the officer in command of the Beckett's Park institution, asked Mr. Lupton, as chairman of the Leeds General Infirmary, to supply a considerably increased number of beds for the wounded. When it became clear that these could not be provided without turning out civilian patients, the difficulties of having wounded in buildings apart from existing hospitals, and the increased work for the staffs which would be thus entailed, led to the plan of extending existing accommodation. An influential committee was then formed, after an interview with Sir Alfred Keogh, and it was proposed to the War Office that the committee would try to raise in Leeds sufficient money to build a hospital of 750 beds, on condition that the Army authorities would equip and maintain it. This offer was accepted by the Army Council. The new buildings—on the military plans for hutted hospitals—will be built of wood, adjacent to the hospital at Beckett's Park. Apart from kitchens and mess rooms, twelve blocks are suggested. A bridge will connect the new buildings with the existing hospital. Estimates are for £25,000 for the building and £10,000 for the equipment—which last the War Office will provide. The Lord Mayor concluded a powerful and instructive address by remarking that the reason why a city fund was asked for to achieve a purpose which should be paid for out of national resources, was simply that if the people of Leeds did not do this work it would be done too late, and wounded would arrive with the necessary beds not available. The need for the additional beds, he said, was beyond dispute. Before the meeting, the Lord Mayor reported, £17,000 had been promised.

### THIS WEEK'S DRUG MARKET.

Business in the drug market has been quieter during the week, and will not become fully active until the beginning of the New Year. Changes in values have been fairly numerous, but not of outstanding importance. Salicylic acid and sodium salicylate are again dearer, being now fully twenty times the pre-war price. Acetanilide is also dearer. Bromides are still fetching extremely high prices, and the tendency of values continues to be in an upward direction. The value of opium is well maintained. Ipecacuanha has again advanced in price. Eucalyptus oil is in good demand and dearer. Gentian root is extremely scarce.

